# Mounier-Kuhn syndrome: a typical case including CT and bronchoscopic imaging

**DOI:** 10.1093/omcr/omad123

**Published:** 2023-11-28

**Authors:** N Zaghba, Z Laklaai, K Chaanoun, H Benjelloun, N Yassine

**Affiliations:** Respiratory Diseases Department, Ibn Rochd University Hospital, Casablanca, Morocco; Respiratory Diseases Department, Ibn Rochd University Hospital, Casablanca, Morocco; Respiratory Diseases Department, Ibn Rochd University Hospital, Casablanca, Morocco; Respiratory Diseases Department, Ibn Rochd University Hospital, Casablanca, Morocco; Respiratory Diseases Department, Ibn Rochd University Hospital, Casablanca, Morocco

A 67-year-old woman was admitted to our department presenting with a persistent cough characterized by mucopurulent expectoration and dyspnea on exertion, which had been progressively worsening over the course of one week. She also exhibited accompanying fever symptoms. Notably, she shared a history of recurrent lower respiratory infections spanning the last two decades. Upon the initial physical examination, the patient displayed stable hemodynamic and respiratory conditions, albeit with a febrile state reaching 38.5°C.

The patient’s CT scan findings revealed significant dilatation of the trachea and both main left and right bronchi, measuring 32 mm, 20 mm, and 17.5 mm in transverse diameter, respectively (Fig. 1a). These observations served as crucial evidence to confirm the diagnosis of Mounier-Kuhn Syndrome (MKS).

**Figure 1:**
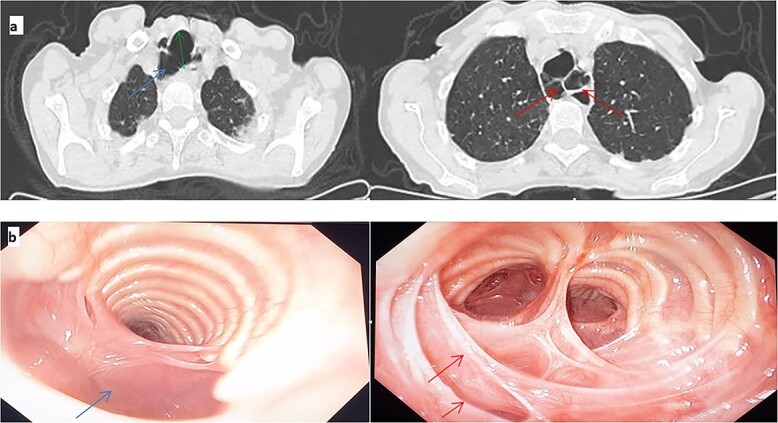
(a) Thoracic CT scan from the upper sections, a marked dilatation of the trachea with an estimated AP (anteroposterior) diameter of 32 mm (green double arrows) and tracheal diverticula (blue arrows) and septa (red arrows). (b) Bronchoscopy. Dilated trachea, diverticula (blue arrows) and septa (red arrows).

MKS is characterized in men by an enlargement of the trachea greater than 25 mm in the transverse section and greater than 27 mm in the sagittal section with diameters of the right and left main bronchi exceeding 18 mm and 21 mm respectively. The measurements defining the condition in women are 21 mm in the transverse section, 23 mm in the sagittal section, 17.4 mm right main bronchi, and 19.8 mm in the left main bronchi [1].

The exact cause of MKS remains elusive, but it appears that atrophy or absence of smooth muscle cells and elastin fibers in the tracheobronchial tree contribute to the development of tracheobronchomegaly, a hallmark of the syndrome [2].

The patient’s history should be investigated for pulmonary tuberculosis, sarcoidosis, cystic fibrosis, or diffuse pulmonary fibrosis, which can lead to pulmonary fibrosis of the upper lung zones and cause acquired tracheobronchomegaly [3]. During bronchoscopy, an increase in the diameter of the trachea and bronchi can be noticed, as well as the confirmation of tracheomalacia (loss of airway structural integrity leading to dynamic collapse) and the presence of diverticula [4]. This procedure was performed and showed a dilated trachea with the presence of diverticula and septa in all bronchial trees (Fig. 1b).

The treatment of Mounier-Kuhn syndrome is generally supportive. Infections should be treated with antibiotics, and mucolytics together with bronchopulmonary hygiene can be considered in patients who need assistance with the elimination of secretions. Prophylaxis with appropriate vaccinations is recommended [5]. Endotracheal stenting has been performed in severe cases [6].

We treated the exacerbation in our patient with antibiotics, physiotherapy, and oxygen therapy. The patient was then discharged with a prescription for influenza and pneumococcal vaccinations, vitamin therapy, and a referral to a physiotherapist to prevent similar episodes.

## Data Availability

All relevant data are within the manuscript.
